# Lithium Treatment Induces Cardiac Dysfunction in Mice

**DOI:** 10.3390/ijms242115872

**Published:** 2023-11-01

**Authors:** Serena L’Abbate, Giuseppina Nicolini, Sabrina Marchetti, Gianpiero Forte, Elisa Lepore, Virginia Unfer, Claudia Kusmic

**Affiliations:** 1 Health Science Interdisciplinary Center, Scuola Superiore Sant’Anna, 56124 Pisa, Italy; serena.labbate@santannapisa.it; 2 Institute of Clinical Physiology, National Research Council (CNR), 56124 Pisa, Italy; giuseppina.nicolini@cnr.it (G.N.); sabrina.marchetti@cnr.it (S.M.); 3 R&D Department, Lo.Li Pharma, 00156 Rome, Italy; g.forte@lolipharma.it (G.F.); e.lepore@lolipharma.it (E.L.); 4 A.G.Un.Co. Obstetrics and Gynaecology Center, 00155 Rome, Italy; virginia.unfer@icloud.com

**Keywords:** lithium therapy, cardiotoxicity, cardiac function, ERK1/2, lithium pollution, mouse

## Abstract

Lithium (Li) salts are commonly used as medications for bipolar disorders. In addition to its therapeutic value, Li is also being increasingly used as a battery component in modern electronic devices. Concerns about its toxicity and negative impact on the heart have recently been raised. We investigated the effects of long-term Li treatment on the heart, liver, and kidney in mice. Sixteen C57BL/6J mice were randomly assigned to receive oral administration of Li carbonate (*n* = 8) or act as a control group (*n* = 8) for 12 weeks. We evaluated the cardiac electrical activity, morphology and function, and pathways contributing to remodelling. We assessed the multi-organ toxicity using histopathology techniques in the heart, liver, and kidney. Our findings suggest that mice receiving Li had impaired systolic function and ventricular repolarisation and were more susceptible to arrhythmias under adrenergic stimulation. The Li treatment caused an increase in the cardiomyocytes’ size, the modulation of the extracellular signal-regulated kinase (ERK) pathway, along with some minor tissue damage. Our findings revealed a cardiotoxic effect of Li at therapeutic dosage, along with some histopathological alterations in the liver and kidney. In addition, our study suggests that our model could be used to test potential treatments for Li-induced cardiotoxicity.

## 1. Introduction

Lithium (Li) is an alkali metal naturally found in soil and water. Li has various industrial and commercial applications, such as being a cell additive in electrolytic aluminium production, a catalyst in chemical reactors, an energy source in batteries, and a sanitising agent for swimming pools and hot tubs, as well as in specialised glass and ceramics [1]. Unfortunately, the widespread use of Li in industry and consumer markets since the 1990s has led to significant environmental pollution from Li waste. This emerging ecological issue requires more attention as high Li concentrations can severely threaten the environment and human health [2,3,4,5].

For over 50 years, Li salts have been a mainstay medication in treating psychiatric disorders, particularly for managing mood and bipolar disorders and preventing relapses [6]. Despite the increasing popularity of other antipsychotics and anticonvulsants, Li remains a primary treatment option in neuropsychiatry [7]. Additionally, recent evidence has discovered that Li salts may have a neuroprotective effect on neurodegenerative diseases such as Alzheimer’s [8], leading to continued interest in its clinical use. Unfortunately, therapeutic doses of Li often come with adverse side effects, leading to a narrow therapeutic window (i.e., the range of blood levels in which the drug can be effective without causing harmful toxicity). The recommended plasma levels for human Li treatment should range between 0.8 and 1.2 mEq/L. However, side effects ranging from mild to severe depend on the therapeutic dosage and drug–drug interactions between Li and other co-medications in patients [9]. Short-term use may result in mild to moderate side effects, while chronic use can lead to potentially severe consequences, influencing patient compliance [10,11]. Adverse side effects commonly affect the nervous system, kidney, thyroid function, hormonal–metabolic balance, and skin [12]. Symptoms may include coarse tremors, motor hyperactivity, ataxia, aphasia, fatigue, cognitive impairment, dermatological affections (acne, psoriasis), and weight gain with glucose and lipid metabolic alterations [13]. Additionally, kidney complications such as nephrogenic diabetes insipidus, nephritis, and chronic progressive renal disease may occur [14]. Cardiac side effects have also been documented in the medical literature through case reports at both low therapeutic doses and acute Li intoxication. Li-induced cardiac toxicity can result in electrocardiographic (ECG) alterations, including dysrhythmias, prolonged QT interval, T wave flattening, and the Brugada ECG pattern. In rare cases, it can lead to moderate systolic dysfunction [15], heart attack, and heart arrest [16,17,18,19].

The heart is a crucial organ that cannot regenerate cardiomyocytes. It is essential to focus on Li exposure’s potential cardiac side effects, including impaired function and tissue damage. Indeed, cardiotoxicity has become a common cause for discontinuing treatments for other target organs, such as some anticancer agents, and the withdrawal of certain drugs from the market due to life-threatening side effects on the heart [20]. Nevertheless, there have been a few systematic studies on the impact of Li on the human heart [21,22]. Furthermore, research on Li-induced toxic side effects in the heart and the underlying mechanisms is limited, even in preclinical models. Some rodent studies have measured tissue biomarkers related to oxidative stress [23,24] and evaluated histopathological changes in the heart after exposure to Li [25,26,27]. As far as we know, there has not been a comprehensive non-invasive and in vivo study on the cardiac effects of Li in a mouse model which includes structural and functional cardiac alterations and multiorgan toxicity-induced histopathological changes in the heart, liver, and kidney.

Therefore, this study aims to investigate the heart’s electrical, structural, and mechanical functions and to histopathologically evaluate cardiac, hepatic, and renal tissues in the C57BL/6J mice treated with Li salts for 12 weeks. Specifically, the study will focus on the animal’s cardiac morphology using ultrasounds, electrical activity, and arrhythmogenic susceptibility using ECG, and multiorgan toxicity using histopathology.

## 2. Results

### 2.1. Animal Well-Being and Plasma Li Levels

The enrolled mice were assigned to two groups: (i) one group was treated with Li carbonate at 45 mg/kg (Li-group), and (ii) the other group was untreated (C-group). All the enrolled mice completed the experimental protocol. The statistical analysis showed no difference in body weight between the two groups, either at baseline (T_0_) (26 (2.8) g and 27 (10.5) g in the C-group and Li-group, respectively) or after 12 weeks (T_1_) (30 [6.5] g and 32 (11) g in the C-group and Li-group, respectively). In addition, the significant increase of animals’ body weight in both groups (C-group by 15.5% and Li-group by 18.5%) over 12 weeks (*p* < 0.001, T_1_ vs. T_0_, for both) was consistent with the standard body weight growth curves for mice of the same strain and age. After 12 weeks of Li carbonate intake in their drinking water, the Li-group exhibited a plasma concentration of Li that was over four-fold higher than the control group (1.7 (0.56) mEq/L in the Li-group vs. 0.38 (0.19) mEq/L in the C-group, *p* = 0.002.

### 2.2. Li Administration Impairs Both Systolic Function and Ventricular Repolarisation

**Figure 1:**
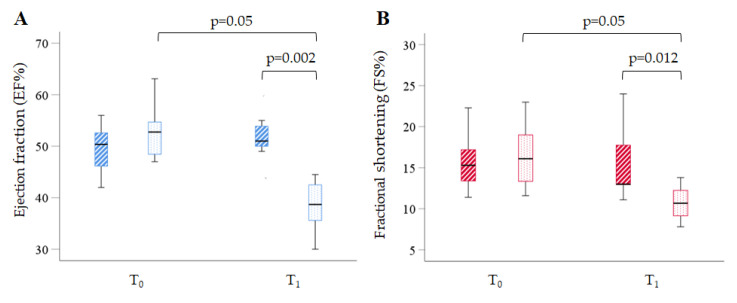
Box plots show the relevant echocardiographic indices at T_0_ and T_1_ in the two groups. (**A**) The percentage of ejection fraction (EF%) in the control (dashed blue boxes) and the Li-treated (dotted blue boxes) groups. (**B**) The percentage of fractional shortening (FS%) in the control (dashed red boxes) and the Li-treated (dotted red boxes) groups. Data are median (IQR), *n* = 8 mice in each group.

Table S1 in the Supplementary presents the results of the high-frequency ultrasound (HF-US) analysis for the C- and Li-groups at T_0_ and T_1_. Upon comparison of all measurements analysed at T_0,_ no differences were found between the two groups, indicating unbiased animal allocation into the groups. However, upon comparison at T_1,_ a few significant differences were observed between the two groups. In general, parameters featuring the diastolic phase of the left ventricular (LV) and chamber volumes remained unaffected by Li administration. Conversely, the systolic function expressed by ejection fraction (EF) and fractional shortening (FS) was significantly lower in the Li-group than in the C-group (*p* = 0.002 and *p* = 0.012 vs. C-group, respectively), as shown in Figure 1.

**Figure 2:**
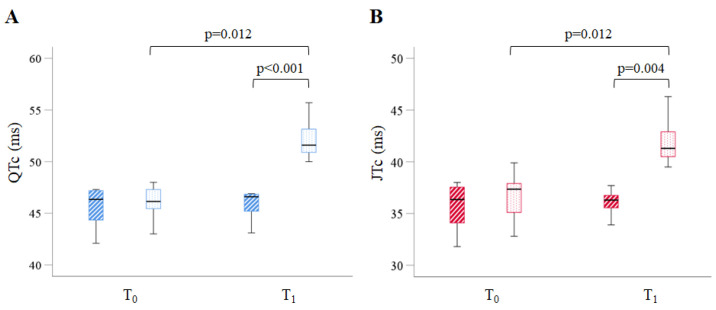
Box plots show the relevant ECG indices at T_0_ and T_1_ in the two groups. (**A**) QT interval corrected for heart rate (QTc) representing the total electrical activity of the LV in the control (dashed blue boxes) and the Li-treated (dotted blue boxes) groups. (**B**) JT interval corrected for the heart rate (JTc) representing the repolarisation time of the LV in control (dashed red boxes) and the Li-treated (dotted red boxes) groups. Data are median (IQR), *n* = 8 mice in each group.

The expected results showed no difference in the longitudinal paired evaluation within the C-group. However, the analysis within the Li-group displayed a significant individual worsening of systolic function (*p* < 0.05 T_1_ vs. T_0_ for both EF and FS). The main ECG metrics measured for the two groups at T0 and T1 are reported in Supplementary Table S2. The analysis showed that 12 weeks of Li intake led to a marked lengthening of total electrical ventricular activity (QT interval corrected for the heart rate, QTc) and repolarisation (JT interval corrected for the heart rate, JTc) time. The QTc and JTc interval values increased by about 14% and 10% compared to the C-group (*p* < 0.001 and *p* < 0.01, respectively) (Figure 2).

There were no significant differences observed in the other measured parameters. The elongation of the JTc, in the absence of notable changes in the QRS interval, suggested that the electrical impairment induced by Li was mainly attributable to the ventricular repolarisation component.

The longitudinal analysis of the C-group did not uncover any significant differences in the examined indices. However, the paired analysis of the Li-group proved that both the QTc and JTc intervals increased significantly at T_1_ compared to T_0_ within the same subjects (*p* = 0.012).

### 2.3. Effect of Li Intake on the Arrhythmogenic Response to β-Adrenergic Stimulation

**Figure 3:**
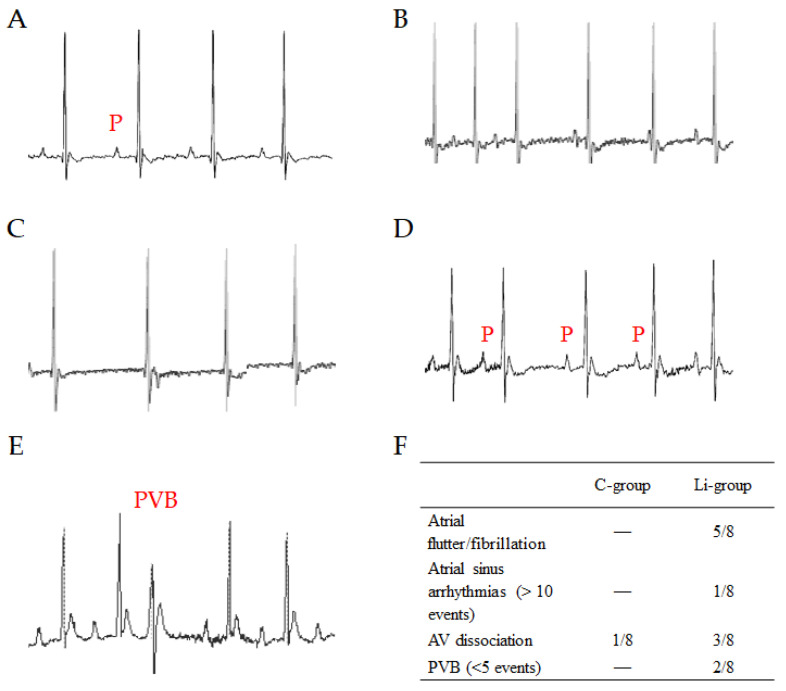
Representative electrophysiological disorders documented by ECG monitoring under adrenergic challenge. Normal sinus rhythm is shown in (**A**). Irregular heartbeat manifestations include atrioventricular dissociation (**B**), atrial fibrillation (**C**), sinus dysrhythmia (**D**), and premature ventricular beat (**E**). P—P wave; PVB—premature ventricular beat. The prevalence of the different arrhythmia events in the two groups is summarised in (**F**).

At T_1_, mice of both the C-group and Li-group underwent serial ECG recordings to examine their arrhythmogenic response to adrenergic stimulation. At baseline, all animals showed regular sinus rhythms and comparable heart rates (513 (99) bpm and 502 (55) bpm in control and Li-treated mice, respectively). After receiving a bolus injection of isoproterenol, heart rate (HR) increased in all mice. The chronotropic response to isoproterenol was similar in both groups, with maximal HR values (657 (29) bpm and 657 (31) bpm) occurring 1 min after drug injection. Heart rates then returned to the physiological range (564 (24) bpm and 555 (23) bpm) 15 min after the drug challenge in the C-group and Li-group, respectively. However, after adrenergic stimulation, over 60% of mice in the Li-group experienced paroxysmal atrial fibrillation and flutter, with a delay of 1–2 min. In addition, after receiving isoproterenol, mice in the Li-group showed various rhythm and conduction disorders such as sinus arrhythmia, premature ventricular beats, and atrioventricular dissociation within 5 to 10 min. These arrhythmic events could occur separately in different mice or, sometimes, together in severe cases. On the other hand, the control mice did not show any atrial fibrillation or flutter, and only one case of atrioventricular dissociation was observed as an abnormality (Figure 3).

### 2.4. The Effect of Li Treatment on Cardiomyocyte Morphometry and Cardiac Tissue

**Figure 4:**
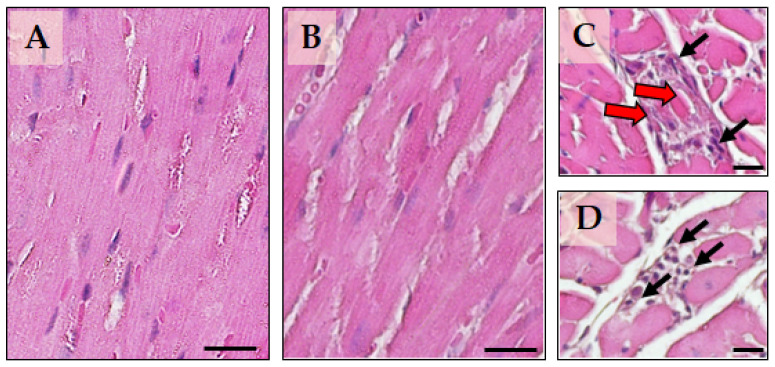
Representative micrographs of myocardial tissue in the two experimental groups. The control group is represented in panel (**A**), while panels (**B**–**D**) represent the group treated with Li. Panels (**A**,**B**) show a longitudinal section of the myocardial tissue, while panels (**C**,**D**) show a transverse section of the myocardial tissue to highlight focal lesions in a few mice of the Li-group. The black arrows indicate the presence of mononuclear cell infiltrates in the cardiac parenchyma. The red arrows point to inflammatory cell aggregates containing degenerated or necrotic fragments of cardiomyocytes. Sections were stained with hematoxylin–eosin. The calibration bar is 20 µm for all panels.

We examined how Li affects the structural remodelling of the heart by investigating cardiac morphology and cardiomyocyte morphometry. Our histopathological analysis at T_1_ showed that the control group had an orderly arrangement of cardiomyocytes (Figure 4A). However, despite a primarily conserved myocardial morphology in the Li group (Figure 4B), some mice experienced moderate damage, including focal or multifocal evidence of cardiomyocytes’ necrosis and the infiltration of mononuclear cells. Such infiltrating cells were compatible with macrophages and occasionally contained damaged cardiomyocyte fragments. In some cases, the infiltrating replaced missing cardiomyocytes and widened the gaps between the remaining cells (Figure 4C,D). We did not find any significant interstitial fibrosis in any of the groups. We measured the LV cardiomyocyte cross-sectional area (CSA); after 12 weeks, Li treatment caused a 25% increase in the area compared to the C-group (277 (56) μm^2^ and 221 (9.8) μm^2^, respectively, *p* = 0.017).

### 2.5. Li Upregulates Hypertrophic Signalling

**Figure 5:**
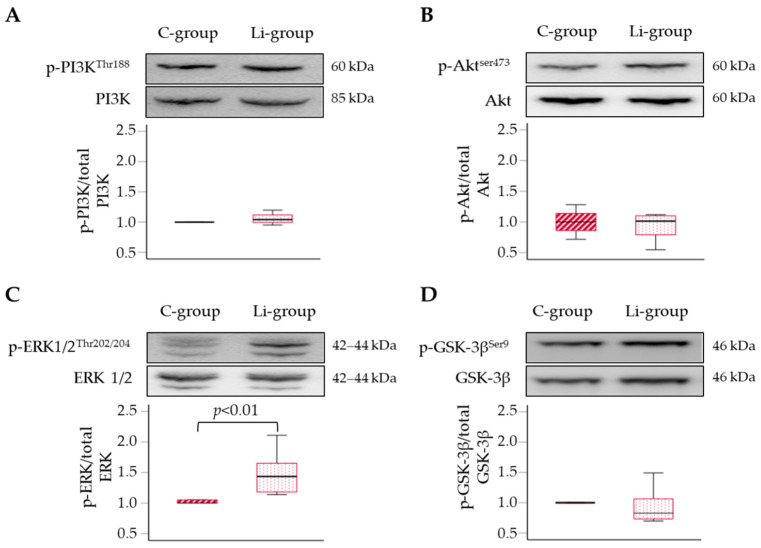
Effects of Li supplementation on the phosphorylation level of four proteins, PI3K (**A**), Akt (**B**), ERK 1/2 (**C**), and GSK-3β (**D**) in the left ventricular myocardium as compared to the control group. Each panel displays representative western blot images of total and phosphorylated proteins and quantifies the phosphorylated ratio to the total level of each protein in the control group (dashed red boxes) and the Li-treated group (dotted red boxes). Data are median (IQR), *n* = 5–7 mice per group.

We further examined the effects of Li treatment on distinct signalling pathways responsible for developing cardiac hypertrophy and ventricular remodelling. We examined the phosphatidylinositol-3 kinase/protein kinase B (PI3K/Akt), extracellular signal-regulated kinase (ERK), and glycogen synthase kinase (GSK)-3β pathways. The total ERK1/2, AKT, and GSK-3β levels, normalised to the reference protein glyceraldehyde-3-phosphate dehydrogenase (GAPDH), were not different in the two tested groups. Then, we normalised the phosphorylation level of each protein to its corresponding total protein level. We found no difference in the phosphorylated to total PI3k, AKT, and GSK-3β ratio between the control and Li-treated mice (Figure 5A,B,D). However, we observed a significant 1.4-fold increased ERK1/2 phosphorylation (*p* < 0.01) (Figure 5C) in the Li group compared with the control group.

These results strongly suggest that Li-induced activation of the ERK pathway occurred by bypassing the PI3K/Akt signalling and was not mediated by GSK-3β inhibition by Ser9 phosphorylation, which is a known downstream target of multiple signalling pathways, including ERK and PI3K/Akt.

### 2.6. The Histopathological Effect of Li on the Liver and Kidney

**Figure 6:**
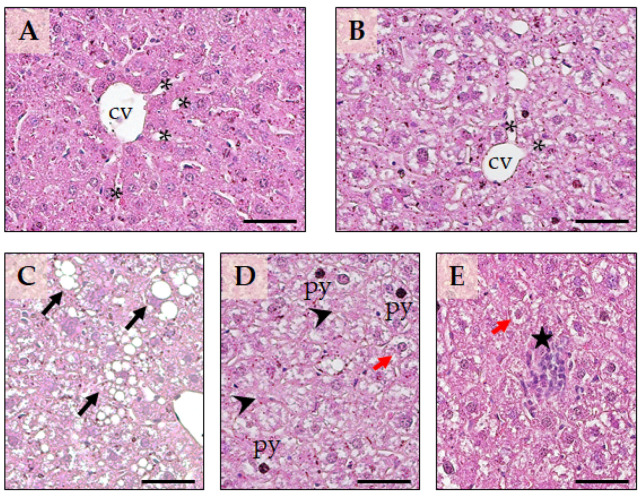
Representative micrographs of the liver architecture of the control group (**A**) and the group treated with Li (**B**). cv—central vein; asterisk—sinusoids. Representative cyto-structural alterations observed in the Li-treated group are represented in (**C**–**E**). The swollen hepatocytes with macrovesicular degeneration are marked with black arrows, while hepatocytes with microvesicular vacuolisation are marked with red arrows. Focal cellular necrosis is indicated with arrowheads, and the inflammatory infiltrate in the liver parenchyma is marked with a black star. Pyknotic nuclei were also observed (py). Sections were stained with hematoxylin and eosin, and the calibration bar for all panels is 50 µm. The images are representative of a total of five–seven animals per group.

Upon histological examination of the liver, the control group displayed the typical structure of hepatic lobules, consisting of tightly packed clusters of clearly defined hepatocytes arranged in a radial pattern around a central vein, interspersed with blood sinusoids. (Figure 6A). However, in the Li group (Figure 6B), we observed noticeable tissue degenerative changes after 12 weeks of drug treatment. Various types of induced lesions were evident and, in some instances, could coexist. In the case of hydropic degeneration, cells become swollen with clear spaces or microvesicular vacuolisation in the cytoplasm, and the nucleus remains centrally located. Hepatocellular micro/macrovesicular vacuolation appears similar to steatosis, with fatty change that can displace the nuclei of the affected cells towards the periphery when extensive. The vacuolisation of hepatocytes, either through hydropic degeneration or fatty changes, was prevalent in all animals. It was primarily diffuse and present from the centrilobular region to the peri-portal area. All animals exhibited focal hepatocellular death, which varied in degree from pycnotic degeneration to coagulative necrosis. Moreover, only a few mice in the Li-group showed sporadic and focal inflammatory cell infiltration, as seen in Figure 6C–E.

**Figure 7:**
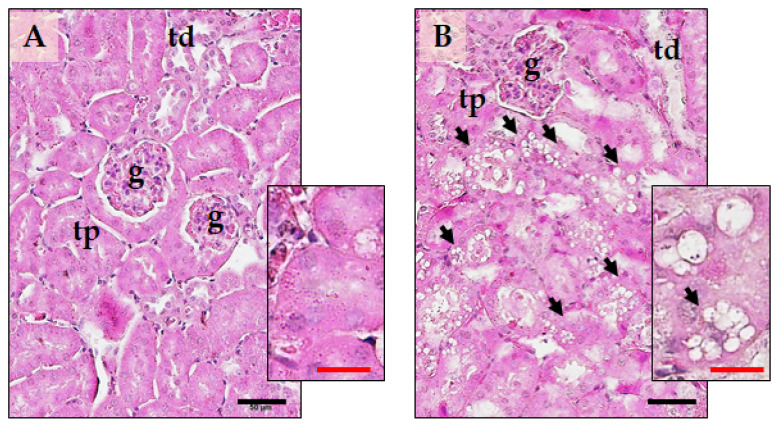
Representative micrographs of the architecture of the parenchyma of the renal cortex in the C-group (**A**) and the Li-group (**B**). The inserts show a higher magnification of the renal parenchyma to highlight its histopathological characteristics. Sections were stained with hematoxylin and eosin. g—renal glomerulus; tp—proximal tubule; td—distal tubule. Black arrows indicate vacuolised epithelial cells. The black calibration bar is 50 µm, while the red calibration bar is 20 µm. The images are representative of a total of five–seven animals per group.

Histopathological investigation of the kidney reported the typical parenchymal architecture of the cortical portion in the control group (Figure 7A). The glomerular structure (g) was well defined, the capsular space (Bowman’s capsule) was proportionate, and the epithelial cell structure in the proximal convoluted tubules (tp) and distal convoluted tubules (td) were normal. However, in the Li group (Figure 7B), we observed vacuolisation and swelling of the tubular epithelial cells (black arrows) after 12 weeks of drug administration. The cellular alterations displayed a variable prevalence among animals and were associated with a modest dilatation of the tubular lumen, mainly affecting the proximal tubules.

Globally, the histopathological lesions found in the liver and kidney aligned with the previous observations of Li-induced organ damage [28,29,30].

## 3. Discussion and Conclusions

In the present study, we provide, for the first time, a broad description of the cardiac structural, electrical, and mechanical alterations induced by the oral administration of Li carbonate for 12 weeks in wild-type mice. We used mice of both sexes but did not find sex differences in Li-induced alterations that would require a stratified analysis of the evaluated parameters.

Our results demonstrated a significant lengthening of LV repolarisation (elongation of the QTc/JTc intervals), reduction of EF, and increased arrhythmogenic response to β-adrenergic stimulation after 12 weeks of Li treatment. Histological analysis found minor myocardial damage and a significant increase in the area of cardiomyocytes in treated animals. Protein expression assays revealed enhanced phosphorylation of ERK1/2 but not of the PI3K/Akt pathway. The Li-induced activation of ERK signalling seems to be not mediated by GSK-3β phosphorylation, one of its canonical downstream targets.

Our investigation has highlighted a lengthening of the QTc/JTc interval. To ensure that the observed differences were not influenced by the possible circadian pattern of QT interval duration [31], we conducted all ECG measurements at consistent time slots. Our findings align with the clinical observations of altered ECG tracings in patients receiving Li treatment [17,32,33,34]. This evidence supports our study’s model and its ability to provide translational insights despite the significant differences in electric currents between human and murine cardiomyocytes. Not surprisingly, Li, an ion that shares similarities with Na and K ions and competes with Ca and Mg ions, can affect the electrophysiological component of cardiac cells. Important arrhythmic events have been signalled in many case reports of patients on Li therapy, mainly associated with acute drug intoxication [17]. In our study, mice treated with Li did not experiment with spontaneous arrhythmic events. However, we observed an evident and significant increase in arrhythmogenic susceptibility in the presence of adrenergic stimulation following Li administration. Atrial arrhythmias were the most common anomalies detected in mice treated with Li, with paroxysmal fibrillation/flutter events prevalent in 62.5% of the cases. Ventricular arrhythmogenesis (extrasystoles) occurred in 25% of the mice treated with Li under adrenergic stimulation. Interestingly, extrasystoles were present in 50% of male mice in the Li group compared to 0% of females. Unfortunately, our sample size became relatively small when stratified by sex for the statistical validation of this observation. However, Liu and colleagues [35] described sex differences in Li-induced ventricular arrhythmogenesis in rabbits. According to the authors, males treated with Li were much more likely to develop Li-induced Brugada-pattern ECG changes with fatal arrhythmia than the treated females. Evidence suggests that sex hormones may significantly impact cardiac rhythm [36]. The possible relationship between Li-induced alterations and sex differences requires further investigations, potentially through a focused study.

Our study has shed light on the previously unknown negative impact of Li treatment on mice’s systolic function, precisely, the ejection fraction. No comparative studies on rodents and only a few case reports on patients with neurological disorders undergoing therapeutic Li treatment demonstrated similar findings [15]. Therefore, the observed alterations in cardiac function induced by Li treatment deserve further investigation.

The presence of cardiac functional alterations induced by treatment with Li in the absence of evident and significant histopathological lesions of the myocardium suggests that, in our study, the dosage and timing of administration of the drug are such as to induce functional rather than structural irreversible alterations, at least those detectable with conventional histology at the cellular level. Indeed, sub-cellular level investigations involving organelles such as mitochondria could help better understand cardiomyocytes’ functional impairment. For instance, a study conducted on isolated mitochondria from rat hearts by Salimi and colleagues [24] found that 75 to 1000 µM Li concentrations caused mitochondrial dysfunction.

Our study did not find evident signs of myocardial necrosis or fibrosis. These observations disagree with previous evidence in rats found by other authors [23,25]. Such a discrepancy could be attributed to the difference in sensitivity to cell damage in the different species and the different dosages used in the other studies. We found that prolonged treatment with Li may lead to myocardial hypertrophy, as observed by the increased CSA of cardiomyocytes in the Li treatment group compared to the control group. Mezni and colleagues [23] found that Li treatment in rats leads to the enlargement of myocardial fibers, which is consistent with our findings. In addition, a clinical case recently reported an association between Li intake and hypertrophic cardiomyopathy [37]. These findings are of topical interest and deserve further investigation to define the possible involved mechanisms. Myocardial hypertrophy is an adaptive response caused by various factors, such as hemodynamic overload, myocardial injury, and vascular disease. However, the specific mechanisms underlying myocardial hypertrophy are still not completely understood. Evidence suggests that different signalling pathways are involved in developing cardiac pathological and physiological hypertrophy. The insulin-like growth factor-1 (IGF-1)/PI3K/Akt cascade and GSK-3β are upregulated and are mainly responsible for physiological hypertrophy [38,39]. On the other hand, the activation of the ERK signalling pathway plays a crucial role in regulating pathological cardiac hypertrophy [40,41,42]. Previous research reported that rats with pathological myocardial hypertrophy have high expression levels of p-ERK in their myocardial tissues [43,44]. In an in vitro model of cardiomyocyte hypertrophy induced by phenylephrine, Mao and colleagues [45] discovered that phosphorylation of ERK1/2 played an essential regulatory role in pathological cell growth.

Our findings demonstrated that Li treatment affects the ERK pathway. Ventricular myocardial tissue in the Li-group showed an increased phosphorylation rate of this protein, an event associated with enhanced activity of this signalling pathway. This outcome supports the cellular hypertrophy observed in these mice compared to the control group. On the other hand, we did not highlight differences in phosphorylation levels and, presumably, the activation of the PI3K/Akt signalling pathway between the control and Li-treated groups. However, it is essential to note that conflicting research findings exist on this topic.

For instance, Lee and colleagues [39] assessed the impact of sub-therapeutic doses of Li on post-infarction ventricular remodelling in rats. The findings indicated that administering a low dose of 1 mmol/kg per day of Li activated the PI3K/Akt cascade without affecting the ERK pathway, even in their Li-treated sham group compared to untreated sham rats. This activation also correlated with the promotion of hypertrophy, which was confirmed by an increase in the CSA of cardiomyocytes [39]. In any case, the authors did not discuss the implications of the results obtained from Li treatment in their sham groups. Therefore, future research will hopefully clarify whether the effects of Li on cardiac hypertrophy and its associated signalling pathways are dose-dependent.

Several studies have demonstrated that blocking the activity of GSK-3β can result in cardiac hypertrophy [46,47], since GSK-3β is crucial in integrating signals from multiple signalling pathways in the myocardium, including ERK and PI3K/Akt. Li has been found to inhibit both GSK-3 isoforms (α and β) both in vitro [48,49] and in vivo [50]. Such inhibition may occur both directly and indirectly. The former implies Li competes with Mg sites due to their similar ionic radius [51,52]; the latter refers to Li-induced increased phosphorylation at Ser9 that inhibits GSK-3β [53,54]. We assessed the activity of GSK-3β by analysing the ratio of Ser9 phosphorylated protein to total protein levels, and our findings indicate no significant difference between the Li and control groups. Noteworthy, the phosphorylation level may only refer to the indirect inhibition. For instance, in a study by Hamstra et al. [55] on murine left ventricles, a subtherapeutic dose of Li was administered over six weeks. The study found that Li feeding reduced GSK3 activity, measured through an enzymatic activity assay. It did not, however, have any impact on the Ser9 phosphorylation level of GSK-3α and GSK-3β isoforms. Overall, although the activity assay cannot distinguish between the two enzyme isoforms, our study did not examine GSK-3 activity in myocardial tissue to conclusively rule out the hypothesis of Li having an inhibitory effect on GSK-3β. Moreover, the absence of variation in the level of Ser9 phosphorylation of GSK-3β contradicts the data obtained by Shen et al. [56] in an in vitro study on human cardiomyocytes. Their study reported Li regulates cardiomyocytes’ functions by controlling GSK-3β signalling activity. However, in their experiment, the effect was observed only when the cells were incubated with concentrations of 2.5 or 5 mmol/L for 48 h, which are difficult to reach in vivo, even in conditions of acute intoxication. Overall, molecular pathways underpinning Li-induced cardiac hypertrophy and the eventual effects depending on different drug dosages require more profound research and investigation.

It is essential to acknowledge some limitations of our study. Our research on the cardiac effects of Li was conducted on a mouse model. However, it is necessary to take further steps to define the potential clinical application and translatability of these findings to human patients undergoing Li therapy. Additionally, we only investigated mice treated solely with Li. To align the study more closely with the clinical setting, it should also be expanded to include a mice population receiving co-medications. This would allow us to evaluate the potential additive adverse effects and the relative impact of drug–drug interactions. Nonetheless, our preclinical study aimed to draw attention to the cardiotoxic effects of lithium, which deserve more attention in humans due to its widespread clinical use as a pharmacological treatment and its impact on environmental pollution.

### Conclusions

After 12 weeks of orally administering Li salts, mice exhibited a significant impairment in LV systolic function and repolarisation. Additionally, they were more prone to arrhythmias under adrenergic stimulation. The Li treatment caused significant cardiomyocyte hypertrophy, but no consistent histopathological lesions occurred in the myocardium. Our findings align with the cardiotoxic impact of long-term therapy with Li in its therapeutic doses. The modulation of ERK1/2 signalling, underlying cardiac hypertrophy, was associated with these effects. Considering the concern regarding Li environmental pollution and the widespread use of such a compound in psychiatric therapies, these results provide new insights into the cardiac side effects of Li. In addition, our evidence paves the way for further research into the mechanisms underlying Li’s cardiac side effects and potential therapeutic interventions to prevent or limit Li-induced cardiotoxicity.

## 4. Materials and Methods

### 4.1. Animals and Experimental Protocol

The study was approved by the Local Ethical Panel and Italian Ministry of Health (Prot. n° 839/2021-PR) and conforms to principles of laboratory animal care demanded by European Directive and Italian laws.

Sixteen C57BL/6 mice (sex ratio 1:1, 10–12 weeks old) were purchased from Envigo Laboratory (Envigo, Udine, Italy). Animals were kept in a controlled environment at a temperature of 21 ± 0.5 °C and humidity of 55 ± 2%), with 12:12 h light–dark cycles. Mice were fed Teklad Global 16% Protein Rodent Diet chow (Envigo, Udine, Italy) and had free access to water. Mice were acclimatised for two weeks before starting the protocol.

At the beginning of the 12-week protocol, mice were divided into two groups (n = 8 each, balanced by sex and age). One group was treated with Li carbonate at 45 mg/kg (Li-group), while the other was untreated (C-group). The mice underwent non-invasive cardiac characterisation at baseline (T_0_) and again after 12 weeks of Li treatment (T_1_), using high-frequency ultrasound (HF-US) imaging and surface ECG recordings. Before each investigation, the mice’s body weights were measured.

At endpoint T_1_, mice were challenged with i.p. isoprenaline, a β_1_-adrenoreceptor agonist, to evaluate their vulnerability to arrhythmias. Afterwards, heart and blood samples were collected during sacrifice for histological analyses and to determine plasma Li levels.

### 4.2. Li Administration

Li treatment involved administering Li_2_CO_3_ (Merck Life Science, Milano, Italy) daily using drinking water at approximately 45 mg/kg/day. Animal dosage was calculated using the formula based on the adult human minimal therapeutic dose of 300 mg/day, according to the following equation:

AED (mg/kg) = Human dose (mg/kg) × K_m_ ratio, (1)

where AED is the animal equivalent dose, and K_m_ is the correction factor estimated by dividing the average body weight (kg) of species by its body surface area (m^2^) [57].

The drug dose was less than 1/10th of the mouse LD50 of 531 mg/kg (according to RTECS, Registry of Toxic Effects of Chemical Substances: National Institute of Occupational Safety and Health, Cincinnati, OH, USA). The amount of Li_2_CO_3_ ingested was determined by monitoring daily water intake.

### 4.3. High-Frequency Ultrasound Examinations

All the animals were imaged using a high-resolution US system (Vevo 3100, FUJIFILM VisualSonics Inc., Toronto, ON, Canada) under gaseous anaesthesia (1.5% *v*/*v*, preceded by induction at 3% *v*/*v* in pure oxygen, at 1 L/min flow rate). Briefly, mice were placed supine on a temperature-controlled board with ECG electrodes for heart rate recording. Body temperature was monitored via a rectal probe using the Vevo 3100 Physiological Monitoring Unit (FUJIFILM VisualSonics Inc., Toronto, ON, Canada). The left ventricle (LV) images were acquired using B-mode modality with a 40 MHz probe (MX 550S, FUJIFILM VisualSonics Inc., Toronto, ON, Canada) in parasternal long-axis and short-axis views. Diastolic function was assessed using pulse wave Doppler in a 4-chamber projection. LV Trace software (version 5.7.1, FUJIFILM VisualSonics Inc., Toronto, ON, Canada) was used to analyse all images offline for cardiac structure and function. Systolic and diastolic LV volumes, stroke volume (SV), ejection fraction (EF), longitudinal fractional shortening (FS), LV mass, and cardiac output (CO) were evaluated. PW Doppler images were used for the evaluation of the diastolic and systolic function according to the following: ratio of the early to late ventricular filling velocities (E/A), isovolumic relaxation time (IVRT), aortic ejection time (Aet), and isovolumic contraction time (IVCT).

### 4.4. Electrocardiographic Recordings and Analysis

The standard ECG lead configuration (i.e., type I, II, III, aVR, aVL, aVF) was used to record surface electrical activity. Needle electrodes were inserted subcutaneously into the limbs of sedated mice (1% isoflurane) while placed on a temperature-controlled board to keep their body temperatures in the physiological range (36.5–37.5 °C). ECG tracings were acquired continuously for 5 min at 4 kHz sampling frequency using Power Lab instrumentation (ML135 PowerLab/8SP) equipped with ML135 Dual Bio Amp amplifier and MLA0112 ECG Lead Switch Box (ADI Instruments Ltd., Oxford, UK). The signals were filtered (50–500 Hz bandpass filter) and digitised (16 bits), and measurements included heart rate, P and QRS morphology, PR interval, QRS duration, rate corrected QT interval (QTc), and JT interval (JTc, an index of ventricular repolarisation). For QTc and JTc calculations, we used the correction equation recommended by Mitchell [58]. This equation is based on the Bazett formula and adapted for mice:

QTc = QT/(RR/100)^1/2^, (2)

where QT is the time from the beginning of the QRS complex (representing ventricular depolarisation) to the end of the T wave (expressive of ventricular repolarisation), and RR is the time elapsed between two successive R-waves of the QRS signal on the electrocardiogram. We conducted ECG measurements in the 8:00–9:30 a.m. interval to eliminate diurnal variations that could impact the individual QT interval duration.

### 4.5. Arrhythmogenic Response to β-Adrenergic Challenge

To compare the arrhythmic response to β-adrenergic stimulation, sedated mice (1% isofluorane in pure oxygen) were challenged with isoproterenol (Isoprenaline Chlohydrate, Monico S.p.A., Venezia, Italy) administered intraperitoneally at a dose of 2 mg/kg of body weight.

ECG output was continuously recorded for 15 min, with baseline ECG recordings starting 5 min before the isoproterenol injection. Based on abnormal QRS waveform, ventricular premature beats were identified. At the same time, analysis of the P wave morphology and PP and RR intervals allowed the diagnosis of impulse formation and conduction disturbances. Atrial fibrillation and flutter were identified based on the absence of a distinguishable single P wave preceding the QRS complex and chaotic disturbance of the isoelectric line. Ventricular arrhythmic events were classified according to the Lambeth Conventions guidelines.

### 4.6. Plasma Determination of Li

Approximately 500 µL of blood was collected in anaesthetised mice via the vena cava using K_2_EDTA microtubes at T_1_. Blood was centrifuged, and the plasma was stored at −20 °C for subsequent analyses. The concentration of free Li in the plasma samples was determined using a colorimetric kit (Lithium assay kit, Abcam, Cambridge, UK).

### 4.7. Histological Analysis Studies

Mouse hearts, liver, and kidney samples were harvested at sacrifice, fixed in buffered 4% formaldehyde, and embedded in paraffin. Heart sections were cut from the ventricle’s midpoint at the papillary muscles’ level and stained for blinded morphometric determination of cardiomyocyte cross-sectional area. Slides of each organ were processed for classical histological (hematoxylin–eosin, HE) and histochemical (Picrosirius red for collagen deposition) stains.

Histological images (20×, 200×, and 400×) were acquired by light microscope (Olympus BX43, Japan) and digitised using an RGB video camera (Olympus DP 20, Japan).

The determination of cardiomyocyte cross-sectional area (CSA) was performed using a magnification of 400× and a digital image analyser (CellSens, Olympus, Tokyo, Japan). Only cardiomyocytes with well-defined cell boundaries and a ratio between the short and long axes < 1.2 were manually delineated to determine CSA. The parameter was calculated in ≥150 cross-sectioned cardiomyocytes per group from 6 different mice, balanced by sex. Cardiomyocyte CSA was expressed in μm^2^.

### 4.8. Protein Extraction and Western Blotting Analysis

Frozen tissue samples of LV (30–50 mg) were pulverised and then homogenised in ice-cold lysis buffer (20 mM Tris-HCl pH 8.0, 20 mM Nacl, 10% glycerol, 1% NP40, 10 mM EDTA, 2 mM PMSF, 2.5 μg/mL leupeptin, 2.5 μg/mL pepstatin) with a Tissue-lyser instrument (Qiagen). The homogenates were centrifuged at 11.000× *g* for 15 min. Supernatants were collected, aliquoted, and stored at −80 °C until use. Protein concentrations were determined by bicinchoninic acid assay (Pierce, Thermo Scientific, Waltham, MA, USA).

LV myocardial extracts (30 µg) were loaded and separated on 4–12% polyacrylamide electrophoresis gels (Bolt Bis-Tris mini gels Life Technologies), then transferred to iBlot 0.2 mm PVDF membranes (Life Technology, Carlsbad, CA, USA). Non-specific protein binding was blocked with 5% nonfat milk in TBS-T at room temperature for one hour. The membranes were incubated overnight at 4 °C with specific rabbit polyclonal antibodies (Cell Signaling Technology Inc., Danvers, MA, USA, dilution 1:1000): against p-AKT(ser473) (#9271), AKT (#9272), p-PI3K(Tyr 199) (#4228), PI3Kp85 (#4292), p-GSK3β (#5558), GSK3β (#12456), p-ERK1/2 (#9101), and ERK1/2 (#9102). After washes, membranes were incubated at room temperature for one hour with secondary anti-rabbit IgG-HRP (#7074, Cell Signaling Technology Inc., Danvers, MA, USA, 1:2000). Proteins were visualised using Amersham ECL™ Western Blotting Analysis System for electrochemiluminescence (ECL) (RPN2109, Amersham, Buckinghamshire, UK). The signal from the ECL substrate was analysed and documented using ALLIANCE-CAPT Advance software (version *16.08a*, UVITEC, Cambridge, UK). Protein expression of the phosphorylated protein was normalised to the total one. The bands of total proteins were normalised to GAPDH (# 2118) (1:5000) expression.

### 4.9. Statistical Analysis

Numeric values are listed as median (interquartile range). For all the parameters evaluated, a two-sided Kolmogorov–Smirnov test for independent samples was used to determine the differences between the C-group and Li-group at each experimental time point. Intra-group variations were assessed using a two-sided Wilcoxon test for paired samples. Tests were considered statistically significant when *p* < 0.05. Data were analysed with SPSS Version 23 (IBM, New York, NY, USA).

## Data Availability

All data generated or analysed during this study are included in this article (and its Supplementary File).

## Supplementary Materials

The supporting information can be downloaded at: https://www.mdpi.com/article/10.3390/ijms242115872/s1.
