# Supplementary material for: Lithium Treatment Induces Cardiac Dysfunction in Mice
Source: Int J Mol Sci. 2023 Nov 1;24(21):15872. doi: 10.3390/ijms242115872 (PMC10650075; doi:10.3390/ijms242115872)
Supplement: Supplementary file 1 [file ijms-24-15872-s001.zip › ijms-2677660-supplementary.pdf]

Supplementary information

Lithium treatment induces cardiac dysfunction in mice

Serena L'Abbate[1], Giuseppina Nicolini[2], Sabrina Marchetti[2,] Gianpiero Forte[3], Elisa Lepore[3], Virginia Unfer[4] and Claudia Kusmic[2*]

[1] Health Science Interdisciplinary Center, Scuola Superiore Sant'Anna, 56124 Pisa, Italy; serena.labbate@santannapisa.it
[2] Institute of Clinical Physiology, National Research Council (CNR), 56124 Pisa, Italy; giuseppina.nicolini@cnr.it (G.N.); sabrina.marchetti@cnr.it (S.M.)
[3] R&D Department, Lo.Li Pharma, 00156 Rome, Italy; g.forte@lolipharma.it (G.F.); e.lepore@lolipharma.it (E.L.)
[4] A.G.Un.Co. Obstetrics and Gynaecology Center, 00155 Rome, Italy; virginia.unfer@icloud.com
* Correspondence: claudia.kusmic@cnr.it; Tel.: +39-0503152669

Ethical Considerations

The study was approved by the Local Ethical Panel and Italian Ministry of Health (Prot. n° 839/2021-PR) and conforms to principles of laboratory animal care demanded by European Directive and Italian laws. The study aimed to investigate the potential adverse effects of administering lithium salts to mice on the electrical function of the heart, cardiotoxicity and liver and kidney alterations. This study was multi-organ, and to fully understand its impact, it could only be conducted in vivo, using the entire morpho-functional model. Currently, therefore, no replacement for the animal model is available. In our experimental design, we opted for non-invasive methods under general anaesthesia, commonly used in human and veterinary clinics, and administered preparations orally to reduce animal manipulation. We monitored the health conditions of the animals daily and chose a species-specific euthanasia method, i.e., anaesthetic overdose, permitted by National and European laws. All procedures were carried out by adequately trained personnel.

Table S1. HF-US **cardiac parameters at T0 and T1**

	C-group		Li-group	
	**T_0**	**T_1**	**T_0**	**T_1**
Vol_s (μl)	44.3 [13.2]	34.8 [23.5]	43.2 [10.3]	36.9 [26.0]
Vol_d (μl)	87.4 [12.8]	77.3 [32.7]	83.3 [27.0]	76.7 [38.8]
SV (μl)	37.5 [9.9]	42,4 [10.1]	38.4 [18.3]	41.6 [14.9]
EF%	49.7 [8.3]	51 [4.9]	53.0 [6.2]	40.0 [7.4] **†
FS%	14.7 [5.0]	13.0 [5.5]	17.3 [5.7]	10.4 [3.9] * †
LVmass (mg)	119.3 [24.6]	113.1 [26.0]	128.8 [46.4]	124.0 [51.7]
CO (ml/min)	14.2 [7.4]	20.0 [5.7]	18.0 [9.7]	17.3 [4.7]
E/A (a.u.)	1.3 [0.5]	1.5 [0.6]	1.6 [0.7]	1.3 [0.3]
IVRT (ms)	15.2 [5.0]	15.0 [72]	15.0 [4.2]	18.1 [6.4]
IVCT (ms)	14.3 [2.1]	14.6 [5.3]	11.1 [3.8]	14.3 [3.7]
A_{et} (ms)	56.2 [10.8]	55.6 [18.2]	51.7 [9.5]	52.9 [10.4]

Values are median [IQR], *n*=8 for each group. Vol_s: systolic volume; Vol_d: diastolic volume; SV: stroke volume; EF%: ejection fraction; FS%: fractional shortening; LVmass: left ventricular mass; CO: cardiac output; E/A: E/A ratio. IVRT: isovolumic relaxation time; IVCT: isovolumic contraction time; A_{et}: aortic ejection time.
*$p<0.05$ and **$p<0.005$ vs C–group at the correspondent time point; †$p<0.05$ for paired comparison vs T_0 within each group.

Table S2. Electrocardiographic characteristics of the two groups at T_0 and T_1

	C-group		Li-group	
	**T_0**	**T_1**	**T_0**	**T_1**
HR (bpm)	528 [87]	513 [101]	527 [68]	515 [100]
P duration (ms)	11.1 [2.0]	10.8 [1.1]	10.8 [2.5]	9.9 [1.4]
PR interval (ms)	36.6 [2.7]	37.0 [8.7]	37.3 [3.8]	41.2 [7.0]
QRS duration (ms)	10.0 [1.0]	9.6 [1.9]	9.9 [1.0]	10.2 [1.3]
QTc interval (ms)	46.4 [3.5]	45.2 [2.0]	46.6 [1.7]	51.4 [2.9] **§
JTc interval (ms)	36.4 [3.9]	37.0 [3.6]	36.4 [1.8]	40.7 [2.9] *§

Values are median [IQR], *n*=8 for each group. HR: heart rate; bpm: beat per minute; P: wave of atrial depolarisation; PR: interval from the beginning of the P wave to the beginning of the QRS complex; QRS: complex of ventricle depolarisation; QTc: QT interval (time of ventricle depolarisation and repolarisation) corrected for HR; JTc: JT interval (time of ventricle repolarisation) corrected for HR. $^*p<0.01$ and $^{**}p<0.001$ vs C–group at the correspondent time point; $§p<0.05$ for paired comparison vs T_0 within each group.
